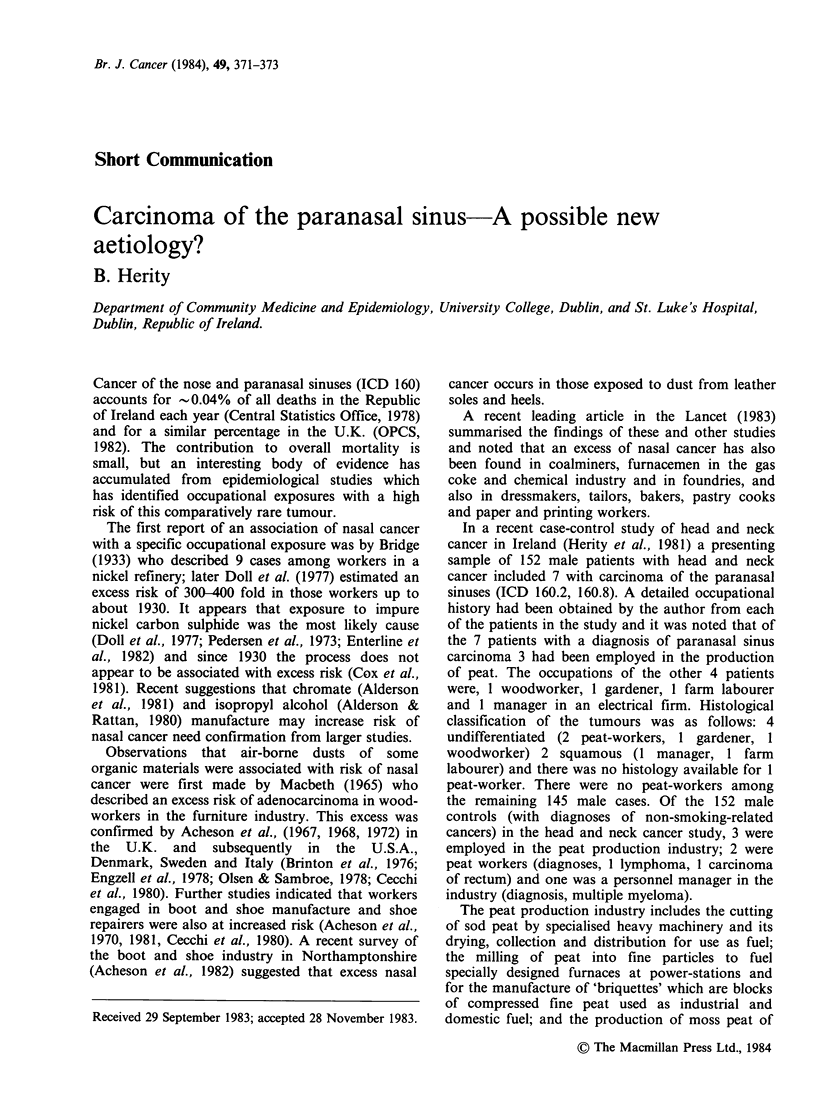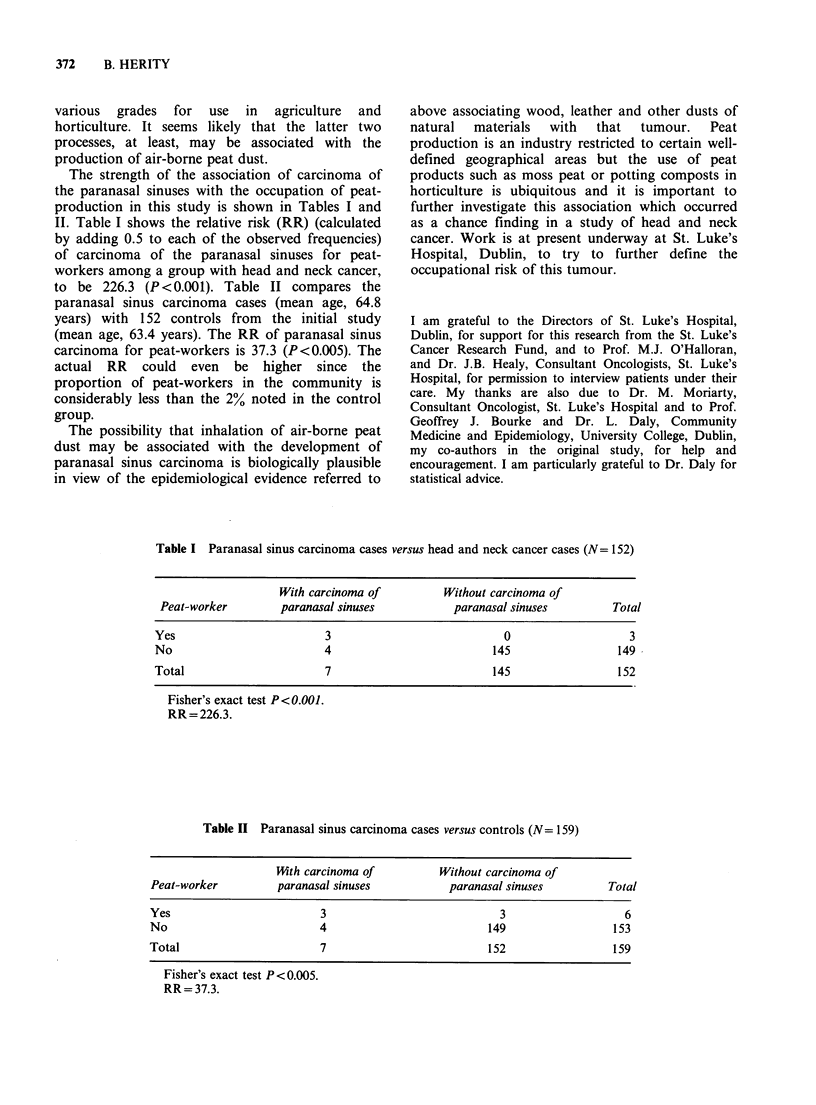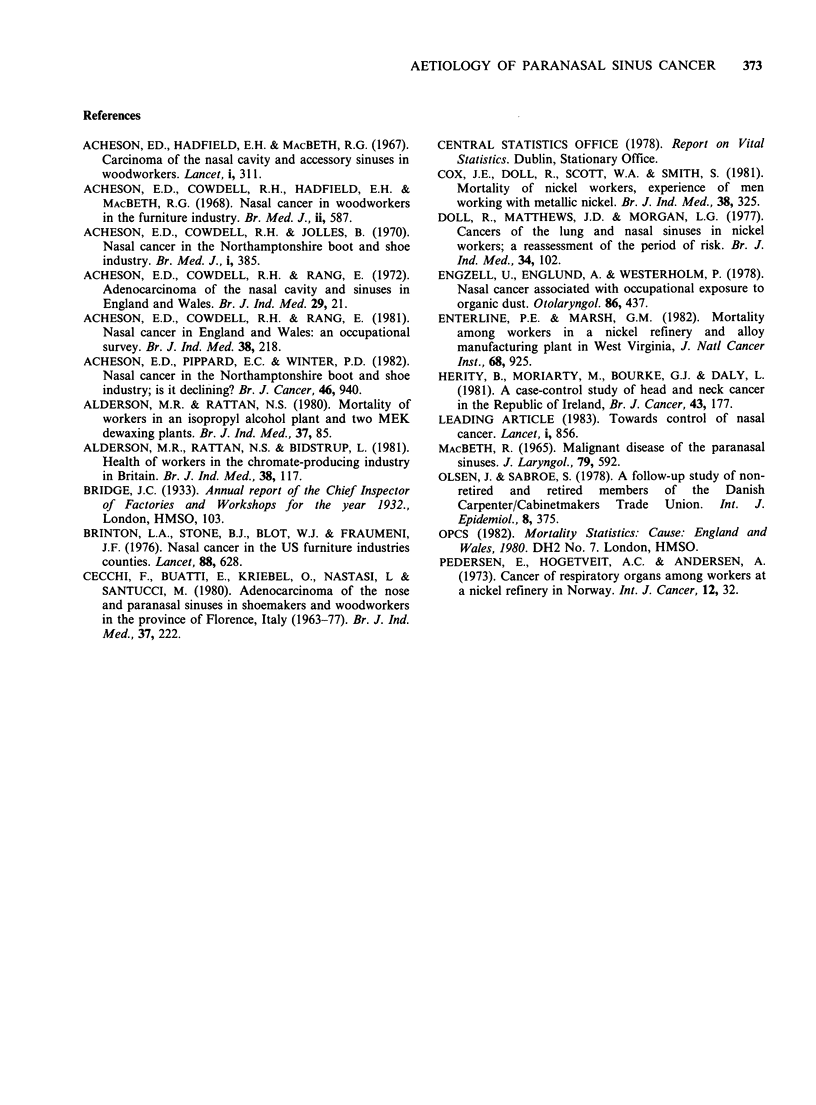# Carcinoma of the paranasal sinus--a possible new aetiology?

**DOI:** 10.1038/bjc.1984.59

**Published:** 1984-03

**Authors:** B. Herity


*Br. J. Cancer* (1984), **49**, 371–373

**Short Communication**

### Carcinoma of the paranasal sinus—A possible new aetiology?

B. Herity

*Department of Community Medicine and Epidemiology, University College, Dublin, and St. Luke's Hospital, Dublin, Republic of Ireland.*

Cancer of the nose and paranasal sinuses (ICD 160) accounts for ~0.04% of all deaths in the Republic of Ireland each year (Central Statistics Office, 1978) and for a similar percentage in the U.K. (OPCS, 1982). The contribution to overall mortality is small, but an interesting body of evidence has accumulated from epidemiological studies which has identified occupational exposures with a high risk of this comparatively rare tumour.

The first report of an association of nasal cancer with a specific occupational exposure was by Bridge (1933) who described 9 cases among workers in a nickel refinery; later Doll *et al.* (1977) estimated an excess risk of 300–400 fold in those workers up to about 1930. It appears that exposure to impure nickel carbon sulphide was the most likely cause (Doll *et al.*, 1977; Pedersen *et al.*, 1973; Enterline *et al.*, 1982) and since 1930 the process does not appear to be associated with excess risk (Cox *et al.*, 1981). Recent suggestions that chromate (Alderson *et al.*, 1981) and isopropyl alcohol (Alderson & Rattan, 1980) manufacture may increase risk of nasal cancer need confirmation from larger studies.

Observations that air-borne dusts of some organic materials were associated with risk of nasal cancer were first made by Macbeth (1965) who described an excess risk of adenocarcinoma in woodworkers in the furniture industry. This excess was confirmed by Acheson *et al.*, (1967, 1968, 1972) in the U.K. and subsequently in the U.S.A., Denmark, Sweden and Italy (Brinton *et al.*, 1976; Engzell *et al.*, 1978; Olsen & Sambroe, 1978; Cecchi *et al.*, 1980). Further studies indicated that workers engaged in boot and shoe manufacture and shoe repairers were also at increased risk (Acheson *et al.*, 1970, 1981, Cecchi *et al.*, 1980). A recent survey of the boot and shoe industry in Northamptonshire (Acheson *et al.*, 1982) suggested that excess nasal cancer occurs in those exposed to dust from leather soles and heels.

A recent leading article in the Lancet (1983) summarised the findings of these and other studies and noted that an excess of nasal cancer has also been found in coalminers, furnacemen in the gas coke and chemical industry and in foundries, and also in dressmakers, tailors, bakers, pastry cooks and paper and printing workers.

In a recent case-control study of head and neck cancer in Ireland (Herity *et al.*, 1981) a presenting sample of 152 male patients with head and neck cancer included 7 with carcinoma of the paranasal sinuses (ICD 160.2, 160.8). A detailed occupational history had been obtained by the author from each of the patients in the study and it was noted that of the 7 patients with a diagnosis of paranasal sinus carcinoma 3 had been employed in the production of peat. The occupations of the other 4 patients were, 1 woodworker, 1 gardener, 1 farm labourer and 1 manager in an electrical firm. Histological classification of the tumours was as follows: 4 undifferentiated (2 peat-workers, 1 gardener, 1 woodworker) 2 squamous (1 manager, 1 farm labourer) and there was no histology available for 1 peat-worker. There were no peat-workers among the remaining 145 male cases. Of the 152 male controls (with diagnoses of non-smoking-related cancers) in the head and neck cancer study, 3 were employed in the peat production industry; 2 were peat workers (diagnoses, 1 lymphoma, 1 carcinoma of rectum) and one was a personnel manager in the industry (diagnosis, multiple myeloma).

The peat production industry includes the cutting of sod peat by specialised heavy machinery and its drying, collection and distribution for use as fuel; the milling of peat into fine particles to fuel specially designed furnaces at power-stations and for the manufacture of 'briquettes' which are blocks of compressed fine peat used as industrial and domestic fuel; and the production of moss peat of

Received 29 September 1983; accepted 28 November 1983.

© The Macmillan Press Ltd., 1984

various grades for use in agriculture and horticulture. It seems likely that the latter two processes, at least, may be associated with the production of air-borne peat dust.

The strength of the association of carcinoma of the paranasal sinuses with the occupation of peat-production in this study is shown in Tables I and II. Table I shows the relative risk (RR) (calculated by adding 0.5 to each of the observed frequencies) of carcinoma of the paranasal sinuses for peat-workers among a group with head and neck cancer, to be 226.3 ($P<0.001$). Table II compares the paranasal sinus carcinoma cases (mean age, 64.8 years) with 152 controls from the initial study (mean age, 63.4 years). The RR of paranasal sinus carcinoma for peat-workers is 37.3 ($P<0.005$). The actual RR could even be higher since the proportion of peat-workers in the community is considerably less than the 2% noted in the control group.

The possibility that inhalation of air-borne peat dust may be associated with the development of paranasal sinus carcinoma is biologically plausible in view of the epidemiological evidence referred to above associating wood, leather and other dusts of natural materials with that tumour. Peat production is an industry restricted to certain well-defined geographical areas but the use of peat products such as moss peat or potting composts in horticulture is ubiquitous and it is important to further investigate this association which occurred as a chance finding in a study of head and neck cancer. Work is at present underway at St. Luke's Hospital, Dublin, to try to further define the occupational risk of this tumour.

I am grateful to the Directors of St. Luke's Hospital, Dublin, for support for this research from the St. Luke's Cancer Research Fund, and to Prof. M.J. O'Halloran, and Dr. J.B. Healy, Consultant Oncologists, St. Luke's Hospital, for permission to interview patients under their care. My thanks are also due to Dr. M. Moriarty, Consultant Oncologist, St. Luke's Hospital and to Prof. Geoffrey J. Bourke and Dr. L. Daly, Community Medicine and Epidemiology, University College, Dublin, my co-authors in the original study, for help and encouragement. I am particularly grateful to Dr. Daly for statistical advice.

**Table I** Paranasal sinus carcinoma cases *versus* head and neck cancer cases ($N=152$)

| *Peat-worker* | *With carcinoma of paranasal sinuses* | *Without carcinoma of paranasal sinuses* | *Total* |
|---|---|---|---|
| Yes | 3 | 0 | 3 |
| No | 4 | 145 | 149 |
| Total | 7 | 145 | 152 |

Fisher's exact test $P<0.001$.

RR = 226.3.

**Table II** Paranasal sinus carcinoma cases *versus* controls ($N=159$)

| *Peat-worker* | *With carcinoma of paranasal sinuses* | *Without carcinoma of paranasal sinuses* | *Total* |
|---|---|---|---|
| Yes | 3 | 3 | 6 |
| No | 4 | 149 | 153 |
| Total | 7 | 152 | 159 |

Fisher's exact test $P<0.005$.
RR = 37.3.

### References

ACHESON, ED., HADFIELD, E.H. & MACBETH, R.G. (1967). Carcinoma of the nasal cavity and accessory sinuses in woodworkers. *Lancet*, **i**, 311.

ACHESON, E.D., COWDELL, R.H., HADFIELD, E.H. & MACBETH, R.G. (1968). Nasal cancer in woodworkers in the furniture industry. *Br. Med. J.*, **ii**, 587.

ACHESON, E.D., COWDELL, R.H. & JOLLES, B. (1970). Nasal cancer in the Northamptonshire boot and shoe industry. *Br. Med. J.*, **i**, 385.

ACHESON, E.D., COWDELL, R.H. & RANG, E. (1972). Adenocarcinoma of the nasal cavity and sinuses in England and Wales. *Br. J. Ind. Med.* **29**, 21.

ACHESON, E.D., COWDELL, R.H. & RANG, E. (1981). Nasal cancer in England and Wales: an occupational survey. *Br. J. Ind. Med.* **38**, 218.

ACHESON, E.D., PIPPARD, E.C. & WINTER, P.D. (1982). Nasal cancer in the Northamptonshire boot and shoe industry; is it declining? *Br. J. Cancer*, **46**, 940.

ALDERSON, M.R. & RATTAN, N.S. (1980). Mortality of workers in an isopropyl alcohol plant and two MEK dewaxing plants. *Br. J. Ind. Med.*, **37**, 85.

ALDERSON, M.R., RATTAN, N.S. & BIDSTRUP, L. (1981). Health of workers in the chromate-producing industry in Britain. *Br. J. Ind. Med.*, **38**, 117.

BRIDGE, J.C. (1933). *Annual report of the Chief Inspector of Factories and Workshops for the year 1932.*, London, HMSO, 103.

BRINTON, L.A., STONE, B.J., BLOT, W.J. & FRAUMENI, J.F. (1976). Nasal cancer in the US furniture industries counties. *Lancet*, **88**, 628.

CECCHI, F., BUATTI, E., KRIEBEL, O., NASTASI, L & SANTUCCI, M. (1980). Adenocarcinoma of the nose and paranasal sinuses in shoemakers and woodworkers in the province of Florence, Italy (1963–77). *Br. J. Ind. Med.*, **37**, 222.

CENTRAL STATISTICS OFFICE (1978). *Report on Vital Statistics*. Dublin, Stationary Office.

COX, J.E., DOLL, R., SCOTT, W.A. & SMITH, S. (1981). Mortality of nickel workers, experience of men working with metallic nickel. *Br. J. Ind. Med.*, **38**, 325.

DOLL, R., MATTHEWS, J.D. & MORGAN, L.G. (1977). Cancers of the lung and nasal sinuses in nickel workers; a reassessment of the period of risk. *Br. J. Ind. Med.*, **34**, 102.

ENGZELL, U., ENGLUND, A. & WESTERHOLM, P. (1978). Nasal cancer associated with occupational exposure to organic dust. *Otolaryngol.* **86**, 437.

ENTERLINE, P.E. & MARSH, G.M. (1982). Mortality among workers in a nickel refinery and alloy manufacturing plant in West Virginia, *J. Natl Cancer Inst.*, **68**, 925.

HERITY, B., MORIARTY, M., BOURKE, G.J. & DALY, L. (1981). A case-control study of head and neck cancer in the Republic of Ireland, *Br. J. Cancer*, **43**, 177.

LEADING ARTICLE (1983). Towards control of nasal cancer. *Lancet*, **i**, 856.

MACBETH, R. (1965). Malignant disease of the paranasal sinuses. *J. Laryngol.*, **79**, 592.

OLSEN, J. & SABROE, S. (1978). A follow-up study of non-retired and retired members of the Danish Carpenter/Cabinetmakers Trade Union. *Int. J. Epidemiol.*, **8**, 375.

OPCS (1982). *Mortality Statistics: Cause: England and Wales, 1980*. DH2 No. 7. London, HMSO.

PEDERSEN, E., HOGETVEIT, A.C. & ANDERSEN, A. (1973). Cancer of respiratory organs among workers at a nickel refinery in Norway. *Int. J. Cancer*, **12**, 32.